# Biochemical Changes and Antioxidant Variations in Date Palm (*Phoenix dactylifera* L.) Varieties during Flower Induction and Development

**DOI:** 10.3390/plants10112550

**Published:** 2021-11-22

**Authors:** Saeed R. H. A. Al Shamsi, Gabriel A. Rabert, Shyam S. Kurup, Mohammed Abdul Muhsen Alyafei, Abdul Jaleel

**Affiliations:** 1 Department of Integrative Agriculture, College of Agriculture and Veterinary Medicine, United Arab Emirates University, Al Ain P.O. Box 15551, United Arab Emirates; 200801575@uaeu.ac.ae (S.R.H.A.A.S.); gabriel.r@uaeu.ac.ae (G.A.R.); skurup@uaeu.ac.ae (S.S.K.); mohammed.s@uaeu.ac.ae (M.A.M.A.); 2 Northern Area Manager in Agriculture Affairs—Technical Services, Agricultural Extension Section, Abu Dhabi Agriculture and Food Safety Authority (ADAFSA), Abu Dhabi P.O. Box 52150, United Arab Emirates; 3 PG & Research Department of Botany, A.V.C. College (Autonomous—Affiliated to Bharathidasan University), Mannampandal, Mayiladuthurai 609 305, Tamilnadu, India

**Keywords:** date palm, enzymes, flowering, antioxidants, flower induction, antioxidant enzymes

## Abstract

The present investigation was carried out to explore the biochemical changes and antioxidant variations, including non-enzymatic and enzymatic antioxidant variations, in the leaves of different varieties of date palm (*Phoenix dactylifera* L.) belonging to the early, mid-, and late-flowering categories in the United Arab Emirates. The changes in the protein and phenol concentration; the ascorbic acid, reduced glutathione, and α-tocopherol contents; and the activity of peroxidase and polyphenol oxidase were studied in the leaves during the preflowering, flowering, and postflowering stages of the date palms. Two varieties each from the early (Shaham, Khanezi), mid- (Barhee, Nabthasaif), and late- (Khasab, Fardh) flowering types were used in this study. The protein content in the leaves was higher in the early flowering varieties during the preflowering stage but lower in the other two varieties. The phenol content showed an opposite trend to the protein. There was significant variation in the ascorbic acid content and a reduction in glutathione and α-tocopherol between the leaves of different varieties. Similarly, the activity of the antioxidant enzyme ascorbate peroxidase in the leaves was higher during the preflowering stage in all varieties. The superoxide dismutase (SOD), polyphenol oxidase (PPO), and catalase (CAT) activity was highest in the Bharhee leaves for all the stages. The peroxidase activity (POD) was highest in the Fardh variety of date palm, whereas the Khanezi variety exhibited the lowest activity. This study can be used as a baseline for developing more protocols for understanding the possible roles of biochemicals, antioxidants, antioxidant enzymes, and their interactions in the regulation of flower development in different date palm varieties.

## 1. Introduction

Date palm (*Phoenix dactylifera*) is mainly cultivated in arid regions, but temperature and photoperiod are important physical parameters causing this tree to initiate flowering. Flowering in this tree is initiated after a temperature fall, and the type of cold period required varies between different varieties [1]. The plant’s blooming is greatly influenced by seasonal changes in environmental factors such as temperature, and a long summer season and a mild winter are required for successful date fruit production [2]. There have been several studies about the flowering- and fruiting-habit changes in date trees [3,4], which are mainly attributed to the antioxidant characteristics of date fruit [5], including the antioxidant capacity, antioxidant compounds, antioxidant enzyme activities in date cultivars during development and ripening [6]. There have also been some recent reports about spontaneous hermaphrodism in female date palms [7]. Enzymatic antioxidant and non-enzymatic antioxidant variations provide an insight into the plausible roles of antioxidants and the activities of antioxidant enzymes in the regulation of flower development in date palm varieties [8].

There are three distinct types of date palm in the UAE: early-, mid-late-, and late-flowering palms [9]. For these three different date palm genotypes, knowledge of the exact physiological/biochemical process of flower induction would be highly beneficial to the production of early-bearing varieties of date palm in the future, which could fetch a very high price in the market by producing fruits early in the season. Investigations into the molecular biology of the flowering process could be supported by the findings of our research. The different physiological factors that influence the flowering behavior of date palm varieties should be explored in order to begin the process of revealing the flower-induction mechanism, as was recently explained in the case of Anemone plants [10]. It is very important to comprehend the character of the enzymes involved in the flower-induction process in date palms, along with the relevant physiological processes. 

Although research has been conducted on the cultivation, production, physiology, and stress tolerance of date palms, there have been no validated reports on the basal mechanism underlying the physiology and/or the flowering behavior of date palms. It is postulated that the antioxidants and hormonal metabolism related to flowering in date palms are enhanced by the activities of peroxidase and polyphenol oxidase, as has been explained in the cases of other plants [11,12]. Studies have even revealed a polyphenol oxidase homolog that is responsible for flower coloration in plants [13]. Moreover, Chin et al. [14] reported an acceleration in flowering in the orchid plant *Oncidium* after the antioxidant status changed, which further proves the role of antioxidants in the flowering of plants. Generally, the transition to flowering is correlated with peroxidase and polyphenol oxidase. Aslmoshtaghi and Shahsavar [15] reported on the biochemical changes involved in olive flower development. It has been stated that the polyphenol oxidase enzyme activity is proportional to the metabolic processes such as vegetative growth and differentiation in plants [16]. In an earlier study, we reported on the variation of antioxidant status in relation to early-, mid-late-, and late-flowering varieties of date palm from the United Arab Emirates [8]. The analysis of the biochemicals and isoenzyme status connected with flowering in early-, mid-late-, and late-flowering varieties of date palm is a useful tool by which to understand the underlying mechanisms. The expected outcome of this experiment was to identify the different physiological factors related to the flowering behavior of date palm varieties, which in turn would be highly significant for the production of early-bearing varieties of date palm in the future. The objectives of this study were to assess the biochemical changes, non-enzymatic antioxidants, and enzymatic antioxidant activities in six date palm varieties in the United Arab Emirates—namely, Shaham and Khanezi (early flowering), Barhee and Nabthasaif (mid-late-flowering), and Khasab and Fardh (late-flowering) in order to identify the different possible physiological factors responsible for flowering.

## 2. Results

### 2.1. Biochemical Contents

**Figure 1:**
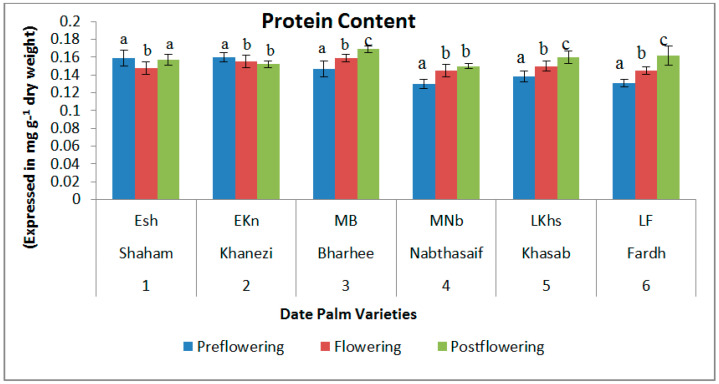
Variations in soluble protein content in date palm (*Phoenix dactylifera*) varieties during different flowering stages. Values are given as the mean ± SD of six experiments for each group. Values that do not share a common superscript (a–c) differ significantly, with *p* ≤ 0.05 (DMRT).

**Figure 2:**
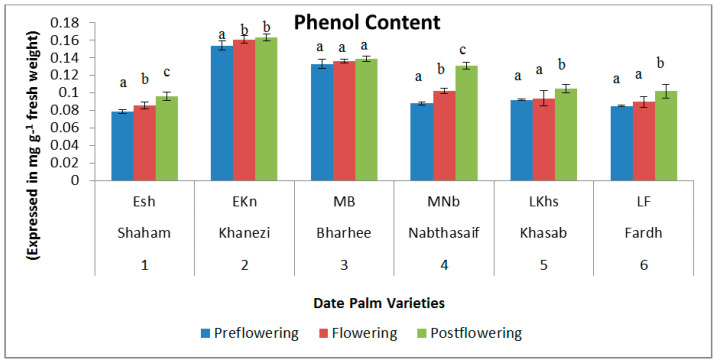
Variations in total phenol content in date palm (*Phoenix dactylifera*) varieties during different flowering stages. Values are given as the mean ± SD of six experiments for each group. Values that do not share a common superscript (a–c) differ significantly, with *p* ≤ 0.05 (DMRT).

The total protein content was high in the early-flowering varieties in the preflowering stage (0.159 and 0.16 mg/g DW, respectively, in Shaham and Khanezi). It gradually decreased in the flowering stage, and then started to rise again in the postflowering stage (Figure 1). In the mid-late- and late-flowering varieties, the protein concentration was low in the preflowering stage. The phenol contents were high during the postflowering stages in all the varieties of date palm (Figure 2). There was an increasing trend of phenol concentration from the preflowering stage to the flowering and postflowering stages. In the mid-late-flowering variety Barhee, there was no significant change in the phenol contents between the flowering periods.

### 2.2. Non-Enzymatic Antioxidants

**Figure 3:**
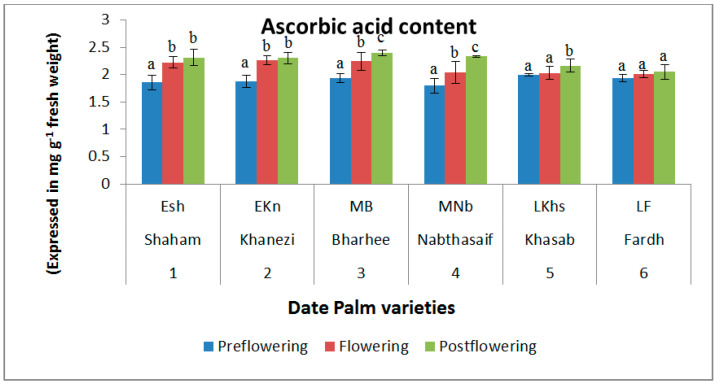
Variations in ascorbic acid content in date palm (*Phoenix dactylifera*) varieties during different flowering stages. Values are given as the mean ± SD of six experiments for each group. Values that do not sharing a common superscript (a–c) differ significantly, with *p* ≤ 0.05 (DMRT).

In all the varieties we studied, the ascorbic acid content was lowest during the preflowering stage. However, the content increased considerably during the flowering stage. There was only a marginal increase in the postflowering period in all the varieties we tested. Among the varieties we studied, the ascorbic acid content was highest in the postflowering stage of Nabthasaif (2.397 mg/g DW), which is a mid-late-flowering variety, and lowest in the preflowering stage of Shaham (1.856 mg/g DW), which is an early-flowering type of date palm. In the flowering phase, the highest ascorbic acid content was recorded in the Khanezi variety (2.262 mg/g DW) and the lowest was recorded in the Fardh variety (2.01 mg/g DW) (Figure 3). 

**Figure 4:**
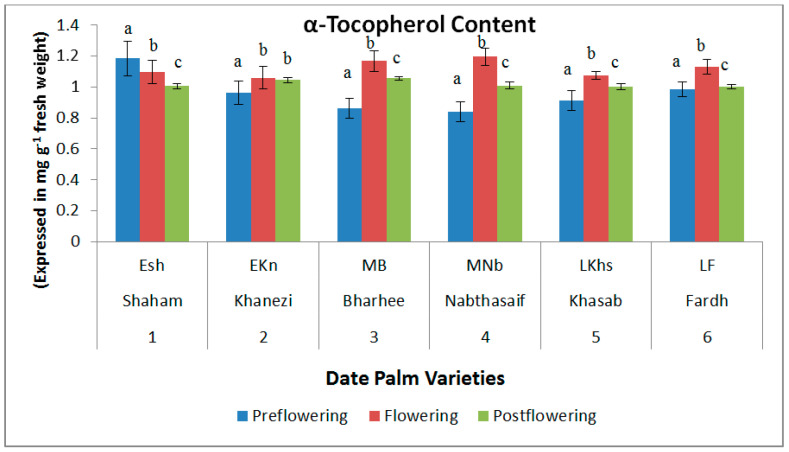
Variations in α-tocopherol content in date palm (*Phoenix dactylifera*) varieties during different flowering stages. Values are given as the mean ± SD of six experiments for each group. Values that do not share a common superscript (a–c) differ significantly, with *p* ≤ 0.05 (DMRT).

**Figure 5:**
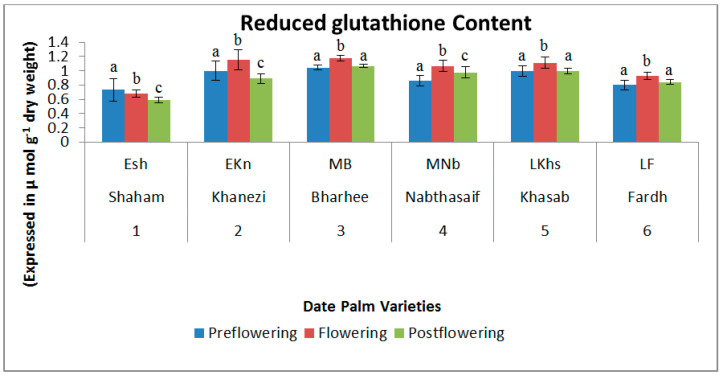
Variations in reduced glutathione (GSH) content in date palm (*Phoenix dactylifera*) varieties during different flowering stages. Values are given as the mean ± SD of six experiments for each group. Values that do not share a common superscript (a–c) differ significantly, with *p* ≤ 0.05 (DMRT).

The concentration of α-tocopherol was high during the flowering period in all six varieties of date palm. However, the content diminished in the postflowering stage, and in the preflowering stage the content was lowest, with the exception of the Shaham variety, which showed the opposite trend. The highest content of α-tocopherol was recorded in the flowering stage of Nabthasaif (1.196 mg/g DW) and the lowest was recorded in the preflowering stage of Nabthasaif (0.838 mg/g DW), which is a mid-late-flowering variety (Figure 4). The content of reduced glutathione showed an increasing trend in the flowering stages of all varieties, but the content was significantly lower in the other two stages of flowering—viz., the preflowering and postflowering stages. The highest reduced glutathione content was recorded in the flowering stage of Khanezi (1.152 mg/g DW), which is an early-flowering variety, and the lowest was recorded in the postflowering stage of Shaham (0.592 mg/g DW) (Figure 5). 

### 2.3. Antioxidant Enzymes

The ascorbate peroxidase activity (APX) was significantly higher in the preflowering stage in all varieties except Nabthasaif. The highest activity was noted in the Barhee variety (1.19 U mg/protein) and the lowest in the Nabthasaif variety (0.659 U mg/protein). The flowering and postflowering stages showed very low activity compared to the preflowering stage (Table 1).

The superoxide dismutase (SOD) activity was highest in the postflowering stage of Barhee (0.597 U/mg protein) and lowest in Fardh (0.295 U mg/protein). Nevertheless, the changes were not significant in any of the varieties between the different flowering seasons (Table 2).

**Figure 6:**
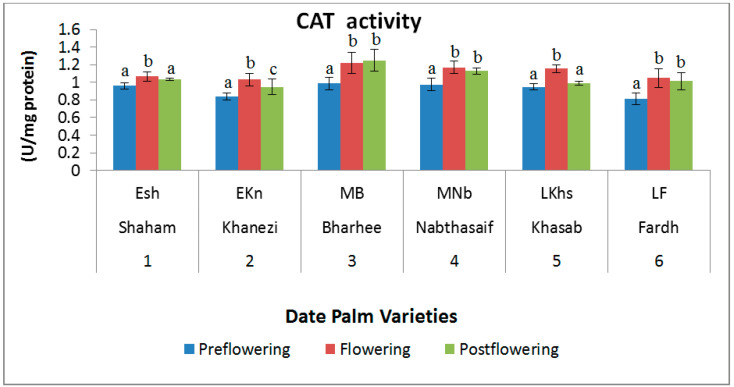
Catalase (CAT) activity in date palm (*Phoenix dactylifera*) varieties during different flowering stages. Values are given as the mean ± SD of six experiments for each group. Values that do not share a common superscript (a–c) differ significantly, with *p* ≤ 0.05 (DMRT).

In all the varieties, the catalase (CAT) activity was high during the flowering season. However, the values decreased slightly during the postflowering period, and reduced further still in the preflowering stage. The maximum value was 1.249 U mg/protein in the postflowering stage of the Barhee variety, and the lowest value was in the preflowering stage of the Fardh variety (0.813 U mg/protein) (Figure 6). In all the studied varieties, the preflowering stage showed the lowest values.

**Figure 7:**
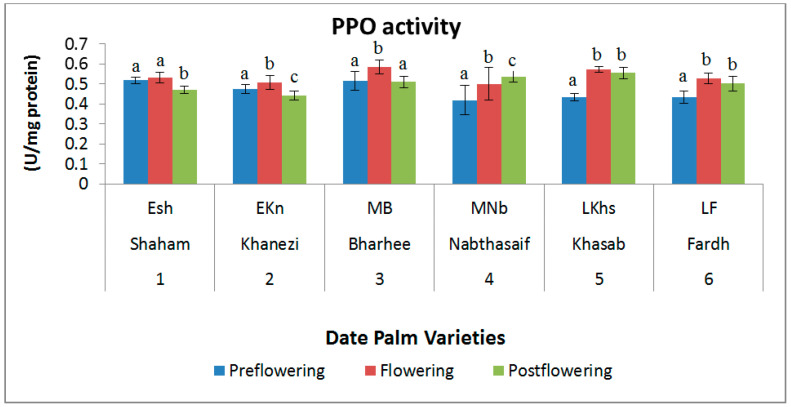
Polyphenol oxidase (PPO) activity in date palm (*Phoenix dactylifera*) varieties during different flowering stages. Values are given as the mean ± SD of six experiments for each group. Values that do not share a common superscript (a–c) differ significantly, with *p* ≤ 0.05 (DMRT).

The polyphenol oxidase (PPO) activity was not significantly altered between the different stages of flowering, except in the Nabthasaif variety, which is a mid-flowering type of palm. A slight increase during the flowering stage was noted, but a decrease during the postflowering and preflowering stages was clear. The highest value of PPO activity was found in Barhee in the flowering stage (0.584 U mg/protein) (Figure 7).

**Figure 8:**
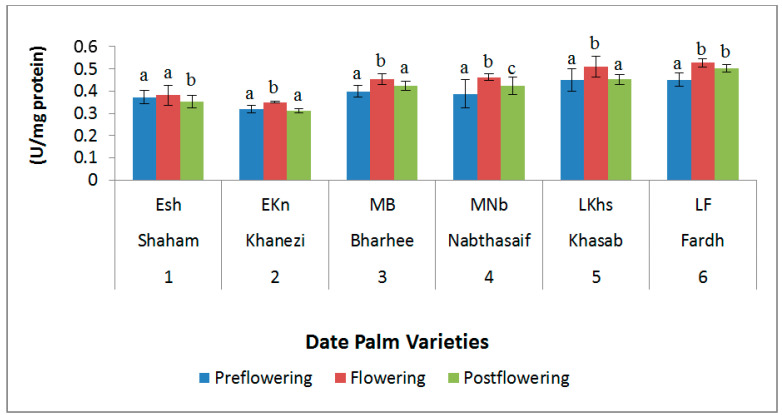
Peroxidase (POD) activity in date palm (*Phoenix dactylifera*) varieties during different flowering stages. Values are given as the mean ± SD of six experiments for each group. Values that do not sharing a common superscript (a–c) differ significantly, with *p* ≤ 0.05 (DMRT).

The peroxidase (POD) activity showed an increasing trend during the flowering stage of the date palm but a decreasing pattern during the preflowering and postflowering stages. The highest values of POD activity were found in the Fardh variety in the flowering stage (0.527 U mg/protein) (Figure 8).

## 3. Discussion

The present investigation was conducted in order to characterize the status of the biochemical content, non-enzymatic antioxidant contents, and enzymatic antioxidant activity in six date palm varieties in the United Arab Emirates. From the results, it was clear that the protein content was high in the early flowering varieties during the preflowering period, which could be attributed to early flowering in these plants. This result was in agreement with reports of the protein involvement in flowering initiation in other plants [17]. Notaguchi et al. [18] explained that proteins such as the FT protein molecules in *Arabidopsis* are critical mobile signals that promote flowering in all varieties, irrespective of the seasons or flowering development. However, the highest contents were noticed in the flowering and postflowering stages in the mid-late- and late-flowering varieties. DuPont and Altenbach [19] reported on the various biochemical factors, including proteins, which influenced grain production in rice. The transition from the vegetative stage to the flowering stage is one of the most important phases in a plant’s life and is triggered by changes in many of the plant’s biochemicals [20]. 

The phenol content was lower during the preflowering and flower-induction stages but higher during the postflowering stage in all the varieties of date palm. The presence of a high amount of phenol may delay flowering and, subsequently, its reduction may induce flowering in plants [21]. This might be due to the presence of phenols in the cytoplasm of leaf cells inhibiting the biosynthesis or transportation of a flowering hormone [22]. Keller and Hrazdina [23] reported an increase in phenol during the postflowering stage of grapes and throughout ripening. Del Baño et al. [24] reported on the role of phenolic diterpenes during the development of plant organs, specifically in the flowers of *Rosmarinus officinalis*.

Seminario et al. [25] observed an increase in the ascorbic acid content of soybean plants under oxidative pressure. The ascorbic acid content of maize showed variation under abiotic stress, suggesting that it played a significant role in the oxidative stress response [26]. In this study, there was an obvious reduction in ascorbic acid in the preflowering stage. Seasonal effects on the ascorbic acid contents of plants in general [27] have been reported on previously. There was a transient rise in the ascorbic acid content which could be seen in the flowering and postflowering stages in all the varieties. Studies suggest that the total endogenous level of AA positively influences the induction of flowering and the accompanying senescence. Both processes require the coordinated regulation of gene expression, which is mediated by various phytohormones such as gibberellins and salicylic acid. It is an established fact that AA acts as a cofactor for the synthesis of GA, which influences the flowering process. Thus, it can be surmised that AA influences phytohormone-mediated signaling during the transition to the flowering phase and during the senescent phase, which explains the rise in AA according to our observations.

The strong antioxidant α-tocopherol induces tolerance to stress [28]. It acts as an antioxidant, preventing free radical peroxidation and injury to cell membranes. Normally, many metabolic alterations take place during flower-bud formation and opening and secondary metabolites due to well defined sequences such as cell division, cellular differentiation, membrane permeability, and cell elongation [29]. The elevation in the α-tocopherol content can be seen to be correlated with the response of the photosynthetic tissues to a variety of abiotic stresses [30]. In the present study, the postflowering stage of the crop overlapped with the summer season, leading to high fluctuations in temperature to protect the plants from the oxidative stress. The elevated levels of α-tocopherol we recorded could be related to the crucial role it played in the inhibition of non-enzymatic lipid peroxidation during the possible stress conditions of the low temperature observed at the flowering stage of the date palms and the high temperature observed at the postflowering stage, coinciding with the onset of summer and resulting in thermal stress.

GSH is a major cell protectant that can directly catch ROS, other oxygen-centered free radicals, and radical centers on DNA or other molecules [31]. In line with the above findings, our results showed depleted GSH levels in the preflowering stage, while in the flowering season the levels were found to be higher than those of other studied varieties. The GSH biosynthesis rate increased due to the stress-inducible activation of glutamylcysteine synthetase at the post-transcriptional level [32], which may have enhanced the stress-associated promotion of flowering. Hence, this finding may benefit the date palm cultivation industry, since flowering is induced only during low-temperature months. Chilling stress is known to cause oxidative stress and to induce changes in the GSH content of plants [33]. This was observed in our study, where noticeable changes in the content of GSH occurred in all the three varieties after chilling treatment lowered the GSH levels and promoted flowering.

Stresses commonly lead to the overproduction of reactive oxygen species (ROS) in plant cells, such as superoxide radicals (O_2_^−^), H_2_O_2,_ and hydroxyl radicals (HO) [34]. Different mechanisms participate in ROS detoxification, and drought results in a significant increase in superoxide dismutase activity [35]. During the flowering and postflowering stages, palms are under stress from the winter and summer seasons, respectively, which might influence their antioxidant metabolism.

There are many reports showing an increase in the activity of APX in plants under many different stress conditions [36]. Hence, a plausible explanation is that the upturns in catalase activity at these stages could have resulted from the accumulation of H_2_O_2_ due to a higher rate of respiration, which in turn might have resulted from the low temperature that is dominant during the flowering season [37]. Superoxide dismutase is the most predominant enzymatic antioxidant found in plant cells [38]. It is a natural scavenger of reactive oxygen species and superoxide radicals and achieves this by combining with active oxygen-free radicals (specifically, superoxide ions) in order to prevent the lipid peroxidation of the cell membrane and damage to the formation of metabolites [39]. 

The combined impact of both antioxidant enzymes (CAT and SOD) is to convert poisonous superoxide radicals (O_2_) and H_2_O_2_ to water and oxygen (O_2_), and, in this manner, to preserve the cells during dry seasons [40]. Likewise, the elevation in SOD activity in the shoots of date palms may be due to their radical scavenging ability. Changes in the activity of antioxidant compounds are signs of plant adaptation to stress conditions [41]. The CAT enzyme showed a significantly high level of activity in the genotype at all intervals and reached its maximum activity level at a moderate level of drought stress [42]. Abassi et al. [42] reported that manganese deficiency was associated with a burst of catalase activity during bud development, which peaked during the fruiting stage of apples, contrary to the results obtained from the mid-flowering variety of date palm used in this study. 

The elevation in CAT activity may be valuable for disproportioning H_2_O_2_, which is key to diminishing senescence under extreme environmental stress conditions [43]. In the peroxisome, the CAT plays a fundamental role in the evacuation of poisonous H_2_O_2_, which is continuously formed during photorespiration by the dismutation of the superoxide radicals generated in the NADH subordinate electron transport system of the peroxisomal layer [44]. The results we obtained for early- and late-flowering date palm varieties were in line with those of Abassi et al. [42], as there was a significant increase in CAT activity in the early-flowering variety at all the three stages, except the late-flowering stage.

During the flowering season, there was no significant difference in PPO activity among all the varieties. However, the POD activity showed a significant increase in all varieties except Shaham. This high POD activity could be a sign that the cold temperature at the preflowering stage caused cold stress, resulting in the production of more reactive oxygen species. POD activity is considered necessary for the oxidation of auxin (IAA) [45], and it has proven necessary for IAA oxidase activity [46]. This has been seen in many plant species. If the POD activity was higher during the preflowering stage, it would exhaust the high IAA necessary for flower induction, so it is sensible to diminish POD activity at first [47]. POD has been shown to perform a function in cell growth and expansion and the synthesis of lignin and suberin [48]. 

## 4. Materials and Methods

The experimental trees were located and marked separately from others at the Al-Foah Research Station of the College of Agriculture and Veterinary Medicine (270° N, 220° S latitude; 510° W, 570° E longitude), UAEU, in Al Ain city, 160 km east from Abu Dhabi, the capital city of the United Arab Emirates. The enzymatic studies and antioxidant content measurements were carried out in three different phases of growth and development—namely, the preflowering, flowering, and postflowering periods. Two varieties from each of the three categories were selected. In each individual variety, three plants were located and marked for analysis. The index leaves were identified in each tree and the leaflets were collected at each stage of flowering for sampling. Samples were collected during the preflowering, flowering, and postflowering stages from these trees. Normal date palm cultivation practices and agriculture procedures were carried out for all the plants under study. The varieties used for the study are given below:

(i) Early season—Shaham (ESh) and Khanezi (EKn);
(ii) Mid-late season—Barhee (MBr) and Nabthasaif (MNb);
(iii) Late season—Khasab (LKh) and Fardh (LFr).

### 4.1. Biochemical Analysis

Protein content was estimated following the standard method [49] and the results were expressed in milligrams per gram of dry weight. The total amount of phenol was estimated by the method of Singleton and Rossi, [50] using gallic acid as the standard.

### 4.2. Non-Enzymatic Antioxidants

Ascorbic acid content was measured as described by Omaye et al. [51] and was expressed in milligrams per gram of dry weight. The α-tocopherol content was measured as described by Baker et al. [52] and calculated using a standard graph made with a known amount of α-tocopherol. Reduced glutathione content was measured as described by Griffith [53]. 

### 4.3. Antioxidant Enzymes

Ascorbate peroxidase (APX, EC: 1.11.1.11) was extracted and its activity level was estimated according to the method of Asada and Takahashi [54]. The extraction of superoxide dismutase (SOD, EC: 1.15.1.1) was carried out using the method of Hwang et al. [55], and its activity was measured as described by Beauchamp and Fridovich [56]. One unit is defined as the amount of change in the absorbance by 0.1 per hour per milligram of protein under the test conditions [57]. The activity of catalase (CAT, EC: 1.11.1.6) was measured as described by Chandlee and Scandalios [58]. Peroxidase (POX, EC 1.11.1.7) was measured by the method described earlier [59]. The activity was expressed in the unit mg^−1^ protein and one unit was defined as the change in the absorbance by 0.1 min^−1^ mg^−1^ protein. The polyphenol oxidase (PPO, EC 1.10.3.1) activity was measured according to the standard method [59]. Similarly to peroxidase, a change in 0.1 absorbance/minute/mg of protein constitutes one unit. For all the enzymatic calculations, the protein content was estimated via the method described in [49], utilizing bovine serum albumin (BSA, Sigma, USA) as the standard.

### 4.4. Statistical Analysis

Statistical analysis was carried out using SPSS 16.0, for all the analyzed parameters. Statistical analysis was performed using one-way analysis of variance (ANOVA), followed by Duncan’s Multiple Range Test (DMRT). The values given are the mean ± SD of six samples for each group. Any *p* values ≤ 0.05 were considered as significant.

## 5. Conclusions

This study provides an insight into the possible roles of biochemicals and antioxidant enzyme activity in the regulation of flower development in date palm varieties. This study explains the possible mechanism behind the early, mid-, and late-flowering patterns of date palm trees. The molecular aspects need to be studied using transcriptomic analysis to understand the upregulation and downregulation patterns of genes in the expression of flowering, and gene analysis needs to be carried out to clearly understand the mechanisms involved in flowering pathways. The present study helps to elucidate the gene expression associated with certain metabolic pathways specific to flowering. In addition, the varietal relations in terms of genetic polymorphism need to be explored in order to gain a further understanding of this phenomenon. Therefore, further studies need to be conducted to validate our conclusions. 

## Figures and Tables

**Table 1:** Ascorbate peroxidase (APX) activity in date palm (*Phoenix dactylifera*) varieties during different flowering stages.

S. No	Variety Name	Category	Flowering Stages		
			Preflowering	Flowering	Post Flowering
1	Shaham	Early Flowering	1.146 ± 0.070 ^a^	0.768 ± 0.033 ^b^	0.794 ± 0.042 ^b^
2	Khanezi	Early Flowering	1.028 ± 0.097 ^a^	0.842 ± 0.026 ^b^	0.985 ± 0.038 ^b^
3	Bharhee	Mid-late Flowering	1.190 ± 0.031 ^a^	0.990 ± 0.040 ^b^	1.000 ± 0.023 ^b^
4	Nabthasaif	Mid-late Flowering	0.832 ± 0.039 ^a^	0.797 ± 0.028 ^a^	0.659 ± 0.063 ^c^
5	Khasab	Late Flowering	1.040 ± 0.074 ^a^	0.796 ± 0.024 ^b^	1.033 ± 0.050 ^a^
6	Fardh	Late Flowering	0.935 ± 0.040 ^a^	0.842 ± 0.064 ^b^	0.802 ± 0.022 ^b^

Values are given as the mean ± SD of six experiments for each group. Values that do not share a common superscript (a–c) differ significantly, with *p* ≤ 0.05 (DMRT).

**Table 2:** Superoxide dismutase (SOD) activity in date palm (*Phoenix dactylifera*) varieties during different flowering stages.

S. No	Variety Name	Category	Flowering Stages		
			Preflowering	Flowering	Post Flowering
1	Shaham	Early Flowering	0.429 ± 0.037 ^a^	0.371 ± 0.019 ^b^	0.300 ± 0.026 ^c^
2	Khanezi	Early Flowering	0.391 ± 0.024 ^a^	0.247 ± 0.017 ^b^	0.302 ± 0.018 ^c^
3	Bharhee	Mid-late Flowering	0.502 ± 0.006 ^a^	0.540 ± 0.026 ^b^	0.597 ± 0.015 ^c^
4	Nabthasaif	Mid-late Flowering	0.333 ± 0.027 ^a^	0.388 ± 0.032 ^b^	0.407 ± 0.026 ^c^
5	Khasab	Late Flowering	0.338 ± 0.027 ^a^	0.311 ± 0.020 ^a^	0.305 ± 0.044 ^a^
6	Fardh	Late Flowering	0.313 ± 0.022 ^a^	0.303 ± 0.044 ^b^	0.295 ± 0.020 ^b^

Values are given as the mean ± SD of six experiments for each group. Values that do not share a common superscript (a–c) differ significantly, with *p* ≤ 0.05 (DMRT).

## Data Availability

Data sharing not applicable.

## References

[1] Zaid A., De Wet P.F. (1999). Chapter IV Climatic requirements of date palm. FAO Plant Prod. Prot. Pap..

[2] Krueger R.R. (2021). Date Palm (*Phoenix dactylifera* L.) Biology and Utilization. The Date Palm Genome.

[3] Al-Ameri A.A., Al-Qurainy F., Gaafar A.R.Z., Khan S., Nadeem M. (2016). Male specific gene expression in dioecious *Phoenix dactylifera* (date palm) tree at flowering stage. Pak. J. Bot..

[4] Masmoudi-Allouche F., Meziou B., Kriaâ W., Gargouri-Bouzid R., Drira N. (2010). In vitro flowering induction in date palm (*Phoenix dactylifera* L.). J. Plant Growth Regul..

[5] Saleh E.A., Tawfik M.S., Abu-Tarboush H.M. (2011). Phenolic contents and antioxidant activity of various date palm (*Phoenix dactylifera* L.) fruits from Saudi Arabia. Food Nutr. Sci..

[6] Awad M.A., Al-Qurashi A.D., Mohamed S.A. (2011). Mohamed. Antioxidant capacity, antioxidant compounds and antioxidant enzyme activities in five date cultivars during development and ripening. Sci. Hortic..

[7] Othmani A., Collin M., Sellemi A., Jain S.M., Drira N., Aberlenc F. (2017). First reported case of spontaneous hermaphrodism in female date palm (*Phoenix dactylifera* L.), cv ‘Alligue’. J. Hortic. Sci. Biotechnol..

[8] Cheruth A.J., Kurup S.S., Subramaniam S. (2015). Variations in hormones and antioxidant status in relation to flowering in early, mid, and late varieties of date palm (*Phoenix dactylifera*) of United Arab Emirates. Sci. World J..

[9] Purayil F.T., Robert G.A., Gothandam K.M., Kurup S.S., Subramaniam S., Jaleel A. (2018). Genetic variability in selected date palm (*Phoenix dactylifera* L.) cultivars of United Arab Emirates using ISSR and DAMD markers. 3 Biotech.

[10] Yari V., Roein Z., Sabouri A. (2021). Exogenous 5-azaCitidine accelerates flowering and external GA_3_ increases ornamental value in Iranian Anemone accessions. Sci. Rep..

[11] Salimonti A., Forgione I., Sirangelo T.M., Puccio G., Mauceri A., Mercati F., Sunseri F., Carbone F. (2021). A complex gene network mediated by ethylene signal transduction tfs defines the flower induction and differentiation in *Olea europaea* L.. Genes.

[12] Ebrahimzadeh H., Abrishamchi P. (2001). Changes in IAA, phenolic compounds, peroxidase, IAA oxidase, and polyphenol oxidase in relation to flower formation in *Crocus sativus*. Russ. J. Plant Physiol..

[13] Nakayama T., Yonekura-Sakakibara K., Sato T., Kikuchi S., Fukui Y., Fukuchi-Mizutani M., Ueda T., Nakao M., Tanaka Y., Kusumi T. (2000). Aureusidin synthase: A polyphenol oxidase homolog responsible for flower coloration. Science.

[14] Chin D.C., Hsieh C.C., Lin H.Y., Yeh K.W. (2016). A low glutathione redox state couples with a decreased ascorbate redox ratio to accelerate flowering in *Oncidium* orchid. Plant Cell Physiol..

[15] Aslmoshtaghi E., Shahsavar A.R. (2016). Biochemical changes involved in self-incompatibility in two cultivars of olive (*Olea europaea* L.) during flower development. J. Hortic. Sci. Biotechnol..

[16] Selvarajan E., Veena R., Kumar N.M. (2018). Polyphenol oxidase, beyond enzyme browning. Microbial Bioprospecting for Sustainable Development.

[17] Lin C. (2000). Photoreceptors and regulation of flowering time. Plant Physiol..

[18] Notaguchi M., Abe M., Kimura T., Daimon Y., Kobayashi T., Yamaguchi A., Araki T. (2008). Long-distance, graft-transmissible action of *Arabidopsis* flowering locus T protein to promote flowering. Plant Cell Physiol..

[19] DuPont F.M., Altenbach S.B. (2003). Molecular and biochemical impacts of environmental factors on wheat grain development and protein synthesis. J. Cereal Sci..

[20] Amasino R. (2010). Seasonal and developmental timing of flowering. Plant J..

[21] Kalra G., Lal M.A. (2018). Physiology of Flowering. Plant Physiology, Development and Metabolism.

[22] Marchiosi R., dos Santos W.D., Constantin R.P., de Lima R.B., Soares A.R., Finger-Teixeira A., Ferrarese-Filho O. (2020). Biosynthesis and metabolic actions of simple phenolic acids in plants. Phytochem. Rev..

[23] Keller M., Hrazdina G. (1998). Interaction of nitrogen availability during bloom and light intensity during veraison. II. Effects on anthocyanin and phenolic development during grape ripening. Am. J. Enol. Vitic..

[24] Del Baño M.J., Lorente J., Castillo J., Benavente-García O., del Río J.A., Ortuño A., Quirin A.K.-W., Gerard D. (2003). Phenolic diterpenes, flavones, and rosmarinic acid distribution during the development of leaves, flowers, stems, and roots of *Rosmarinus officinalis*. Antioxidant activity. J. Agric. Food Chem..

[25] Seminario A., Song L., Zulet A., Nguyen H.T., González E.M., Larrainzar E. (2017). Drought stress causes a reduction in the biosynthesis of ascorbic acid in soybean plants. Front. Plant Sci..

[26] Xiang N., Hu J., Wen T., Brennan M.A., Brennan C.S., Guo X. (2020). Effects of temperature stress on the accumulation of ascorbic acid and folates in sweet corn (*Zea mays* L.) seedlings. J. Sci. Food Agric..

[27] Bilska K., Wojciechowska N., Alipour S., Kalemba E.M. (2019). Ascorbic Acid—The Little-Known Antioxidant in Woody Plants. Antioxidants.

[28] Godoy F., Olivos-Hernández K., Stange C., Handford M. (2021). Abiotic Stress in Crop Species: Improving Tolerance by Applying Plant Metabolites. Plants.

[29] Sood S., Nagar P.K. (2003). Changes in abscisic acid and phenols during flower development in two diverse species of rose. Acta Physiol. Plant..

[30] Upadhyaya D.C., Bagri D.S., Upadhyaya C.P., Kumar A., Thiruvengadam M., Jain S.K. (2021). Genetic engineering of potato (*Solanum tuberosum* L.) for enhanced α-tocopherols and abiotic stress tolerance. Physiol. Plant.

[31] Dorion S., Ouellet J.C., Rivoal J. (2021). Glutathione Metabolism in Plants under Stress: Beyond Reactive Oxygen Species Detoxification. Metabolites.

[32] Hasanuzzaman M., Nahar K., Anee T.I., Fujita M. (2017). Glutathione in plants: Biosynthesis and physiological role in environmental stress tolerance. Physiol. Mol. Biol. Plants.

[33] Chen J., Zhou G., Dong Y., Qian X., Li J., Xu X., Huang H., Xu L., Li L. (2021). Screening of Key Proteins Affecting Floral Initiation of Saffron Under Cold Stress Using iTRAQ-Based Proteomics. Front. Plant Sci..

[34] Sachdev S., Ansari S.A., Ansari M.I., Fujita M., Hasanuzzaman M. (2021). Abiotic stress and reactive oxygen species: Generation, signaling, and defense mechanisms. Antioxidants.

[35] Sarker U., Oba S. (2018). Catalase, superoxide dismutase and ascorbate-glutathione cycle enzymes confer drought tolerance of Amaranthus tricolor. Sci. Rep..

[36] Balfagón D., Zandalinas S.I., Baliño P., Muriach M., Gómez-Cadenas A. (2018). Involvement of ascorbate peroxidase and heat shock proteins on citrus tolerance to combined conditions of drought and high temperatures. Plant Physiol. Biochem..

[37] Zhanassova K., Kurmanbayeva A., Gadilgereyeva B., Yermukhambetova R., Iksat N., Amanbayeva U., Bekturova A., Tleukulova Z., Omarov R., Masalimov Z. (2021). ROS status and antioxidant enzyme activities in response to combined temperature and drought stresses in barley. Acta Physiol. Plant.

[38] Berwal M., Ram C. (2018). Superoxide dismutase: A stable biochemical marker for abiotic stress tolerance in higher plants. Abiotic Biot. Stress Plants.

[39] Yaman S.O., Ayhanci A. (2021). Lipid Peroxidation. Lipid Peroxidation.

[40] Cembrowska-Lech D., Koprowski M., Kępczyński J. (2015). Germination induction of dormant *Avena fatua* caryopses by KAR1 and GA3 involving the control of reactive oxygen species (H_2_O_2_ and O_2_^−^) and enzymatic antioxidants (superoxide dismutase and catalase) both in the embryo and the aleurone layers. J. Plant Physiol..

[41] Campobenedetto C., Mannino G., Beekwilder J., Contartese V., Karlova R., Bertea C.M. (2021). The application of a biostimulant based on tannins affects root architecture and improves tolerance to salinity in tomato plants. Sci. Rep..

[42] Abassi R., Namsi A., Bennasri M., Abdalah H.B., Mâachia S.B., Ouerghi Z., Duran-Vila N. (2014). Manganese deficiency is associated with histological changes in date palm fronds showing brittle leaf disease symptoms. J. Plant Pathol..

[43] Tuzet A., Rahantaniaina M.S., Noctor G. (2019). Analyzing the function of catalase and the ascorbate–glutathione pathway in H2O2 processing: Insights from an experimentally constrained kinetic model. Antioxid. Redox Signal..

[44] Amor N., Jimenez A., Boudabbous M., Sevilla F., Abdelly C. (2019). Implication of peroxisomes and mitochondria in the halophyte Cakile maritima tolerance to salinity stress. Biol. Plant.

[45] Hiraga S., Sasaki K., Ito H., Ohashi Y., Matsui H. (2001). A large family of class III plant peroxidases. Plant Cell Physiol..

[46] Schmidt R., Kunkowska A.B., Schippers J.H. (2016). Role of reactive oxygen species during cell expansion in leaves. Plant Physiol..

[47] Kasraoui M.F., Duquesnoy I., Winterton P., Lamaze T. (2014). Soluble and cell wall bound peroxidase activities are markers of flower bud development stages in lemon (*Citrus limon* L.). J. Appl. Bot. Food Qual..

[48] Liu F., Andersen M.N., Jensen C.R. (2004). Root signal controls pod growth in drought-stressed soybean during the critical, abortion-sensitive phase of pod development. Field Crop Res..

[49] Bradford M.M. (1976). A rapid and sensitive method for the quantitation of microgram quantities of protein utilizing the principle of protein dye binding. Anal. Biochem..

[50] Singleton V.L., Rossi J.A. (1965). Colorimetry of total phenolics with phosphomolybdic-phosphotungstic acid reagents. Am. J. Enol. Vitic..

[51] Omaye S.T., Turnbull J.D., Sauberlich H.E. (1979). Selected methods for the determination of ascorbic acid in animal cells, tissues, and fluids. Methods Enzymol..

[52] Baker H., Frank O., DeAngelis B., Feingold S. (1980). Plasma tocopherol in man at various times after ingesting free or acetylated tocopherol. Nutr. Rep. Int..

[53] Griffith O.W. (1980). Determination of glutathione and glutathione disulfide using glutathione reductase and 2-vinylpyridine. Anal. Biochem..

[54] Asada K., Takahashi M., Kyle D.J. (1987). Production and scavenging of active oxygen in photosynthesis. Photoinhibition.

[55] Hwang S.Y., Lin H.W., Chern R.H., Lo H.F., Li L. (1999). Reduced susceptibility to waterlogging together with high-light stress is related to increases in superoxide dismutase and catalase activities in sweet potato. Plant Growth Regul..

[56] Beauchamp C., Fridovich I. (1971). Superoxide dismutase: Improved assays and an assay applicable to acrylamide gels. Anal. Biochem..

[57] Cherry J.H. (1963). Nucleic acid, mitochondria, & enzyme changes in cotyledons of peanut seeds during germination. Plant Physiol..

[58] Chandlee J.M., Scandalios J.G. (1984). Analysis of variants affecting the catalase developmental program in maize scutellum. Theor. Appl. Gen..

[59] Kumar K.B., Khan P.A. (1982). Peroxidase and polyphenol oxidase in excised ragi (*Eleusine coracana* cv. PR 202) leaves during senescence. Indian J. Exp. Bot..

